# 544. Using Active Surveillance to Identify Monoclonal Antibody Candidates Among COVID-19 Positive Veterans, Atlanta VA Healthcare System

**DOI:** 10.1093/ofid/ofab466.743

**Published:** 2021-12-04

**Authors:** Alexander T Paras, Kathryn E DeSilva, Nora T Oliver, Lauren H Epstein, Nadine M Harris, Emily J Cartwright, Abeer Moanna

**Affiliations:** 1 Emory University School of Medicine, Decatur, Georgia; 2 Atlanta VA Medical Center, Decatur, Georgia; 3 Veterans Administration Hospital, Decatur, Georgia; 4 Emory University School of Medicine, Atlanta, Georgia

## Abstract

**Background:**

Monoclonal antibody (Mab) infusions have reduced hospitalization and mortality among higher risk patients with mild to moderate COVID-19 symptoms. Using an interdisciplinary team approach, we created a clinical team to proactively screen and outreach patients with COVID-19 to equitably offer Mab.

**Methods:**

From December 28, 2020 - May 3, 2021, a clinical team consisting of an Infectious disease pharmacist and physician, reviewed each outpatient with a positive SARS-CoV-2 PCR test at the Atlanta VA Healthcare System (AVAHCS) daily. The clinical team used the published Emergency Use Authorization criteria to determine eligibility. Eligible patients were prioritized using the Veterans Health Administration (VACO) Index for COVID-19 Mortality, which estimates the risk of 30-day mortality after COVID-19 infection using pre-COVID-19 health status (Figure 1). Eligible patients were contacted via telephone to confirm eligibility and obtain verbal consent. We performed SARS-CoV-2 IgG antibody tests when possible prior to Mab infusion, but results did not preclude Mab receipt. Telehealth follow-up occurred at 1- and 7-days post infusion.

Figure 1. Veterans Health Administration COVID-19 (VACO) Index for COVID-19 Mortality

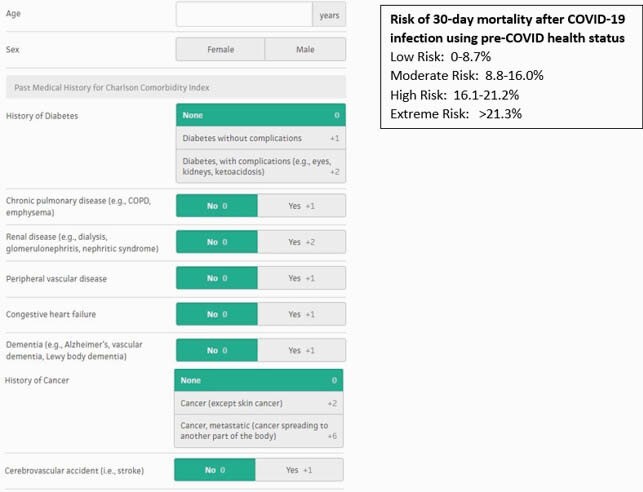

Overview of the elements of the VACO index, part 1 of 2.

Figure 1 continued. Veterans Health Administration COVID-19 (VACO) Index for COVID-19 Mortality

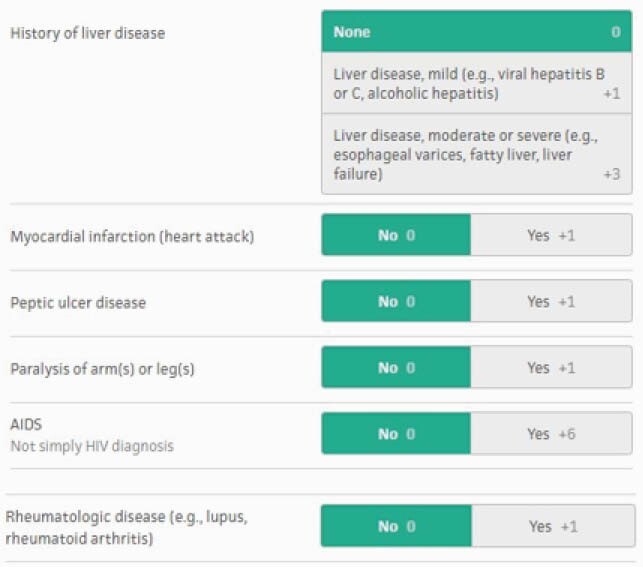

Overview of the elements of the VACO index, part 2 of 2.

**Results:**

In total, 1,346 COVID-19 patients were identified; 86 (6%) patients were eligible, and 48/86 (55%) received Mab infusions (Figure 2). The median time from symptom-onset to positive COVID-19 PCR test result was 6 days (0-9) and the median time from positive COVID-19 PCR test result to Mab infusion was 2 days (0-8). SARS-CoV-2 IgG antibodies were detected in 4 of 24 (17%) patients tested. The most common comorbidities were hypertension (73%) and diabetes, (42%) (Table). Five (10%) patients required hospitalization for worsening COVID-19 symptoms post infusion. No deaths occurred.

Figure 2. Overview of COVID-19 Monoclonal Antibody (Mab) infusion Process

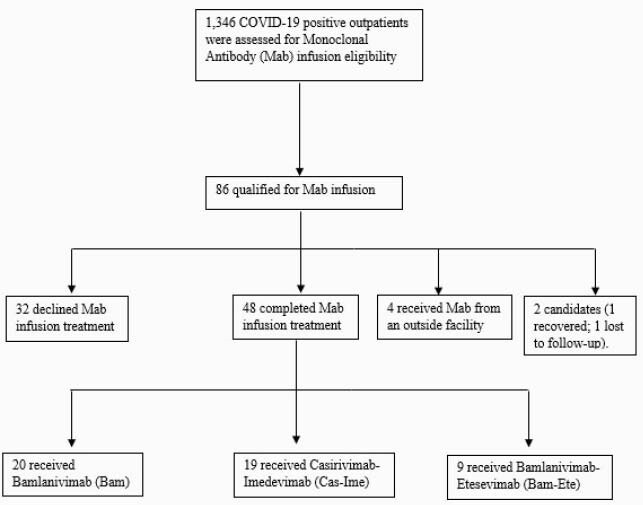

Summary of Mab Infusion Screening Process

Table. Patient Characteristics of Monoclonal (Mab) Infusion Recipients (N = 48)

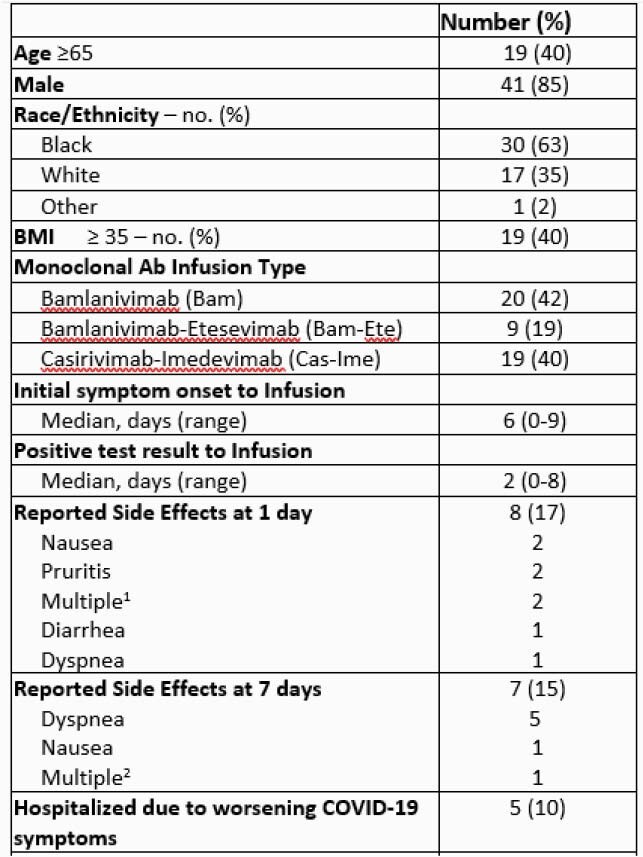

Descriptive Statistics and Findings of Study Data, part 1 of 2

Table continued. Patient Characteristics of Monoclonal (Mab) Infusion Recipients (N = 48)

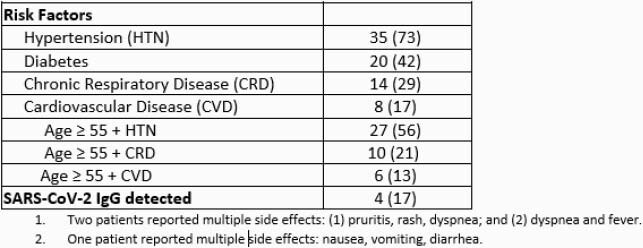

Descriptive Statistics and Findings of Study Data, part 2 of 2

**Conclusion:**

This approach of combining laboratory surveillance and active screening minimized delay in symptoms onset to Mab infusion, thereby optimizing outpatient treatment of COVID-19 disease. Our approach successfully treated a more diverse patient population compared to clinical trials. Mab infusions overall was well tolerated with few hospitalizations and no deaths in this cohort.

**Disclosures:**

**All Authors**: No reported disclosures

